# Improving image reconstruction to quantify dynamic whole-body PET/CT: Q.Clear versus OSEM

**DOI:** 10.1186/s40658-025-00736-5

**Published:** 2025-03-27

**Authors:** Sam Springer, Jeremy Basset-Sagarminaga, Tineke van de Weijer, Vera B. Schrauwen-Hinderling, Walter H. Backes, Roel Wierts

**Affiliations:** 1 Department of Radiology and Nuclear Medicine, Maastricht University Medical Centre, Maastricht, The Netherlands; 2 Department of Nutrition and Movement Sciences, Maastricht University, Maastricht, The Netherlands; 3 Research Institute for Mental Health & Neuroscience and Cardiovascular Diseases, Maastricht University, Maastricht, The Netherlands; 4 Institute for Nutrition and Translational Research in Metabolism, Maastricht University, Maastricht, The Netherlands

**Keywords:** PET/CT, ^18^F-FDG, Reconstruction, Bayesian, Dynamic, Whole-body

## Abstract

**Background:**

The introduction of PET systems featuring increased count rate sensitivity has resulted in the development of dynamic whole-body PET acquisition protocols to assess ^18^F-FDG uptake rate (${K}_{i}$) using ^18^F-FDG PET/CT. However, in short-axis field-of-view (SAFOV) PET/CT systems, multiple bed positions are required per time frame to achieve whole-body coverage. This results in high noise levels, requiring higher ^18^F-FDG activity administration and, consequently, increased patient radiation dose. Bayesian penalized-likelihood PET reconstruction (e.g. Q.Clear, GE Healthcare) has been shown to effectively suppress image noise compared to standard reconstruction techniques. This study investigated the impact of Bayesian penalized-likelihood reconstruction on dynamic whole-body ^18^F-FDG PET quantification.

**Methods:**

Dynamic whole-body ^18^F-FDG PET/CT data (SAFOV PET Discovery MI 5R, GE Healthcare) of healthy volunteers and one lung cancer patient, consisting of a ten-minute dynamic scan of the thoracic region followed by six whole-body passes, were reconstructed with Q.Clear and Ordered Subset Expectation Maximization (OSEM) according to EARL 2 standards. Image noise in the measured time-activity-curves (TAC) was determined for the myocardium, hamstring, liver, subcutaneous adipose tissue and lung lesion for both reconstruction methods. ${K}_{i}$ values were calculated using Patlak analysis. Finally, bootstrapping was used to investigate the effect of image noise levels on ${K}_{i}$ values (bias and precision) as a function of magnitude of ${K}_{i}$ and volume-of-interest (VOI) size for both computationally simulated TACs (${K}_{i}$ = 1.0–50.0·10^–3^·ml·cm^−3^·min^−1^) and the measured TACs.

**Results:**

Compared to OSEM, Q.Clear showed 40–55% lower noise levels for all tissue types (*p* < 0.05). For the measured TACs no systematic bias in ${K}_{i}$ with either reconstruction method was observed. ${K}_{i}$ precision decreased with decreasing VOI size, with that of Q.Clear being superior compared to OSEM for small VOIs of 0.56 cm^3^ in all tissues (*p* < 0.05), with the largest difference in relative precision for small values of ${K}_{i}$. The simulated TACs corroborated these results, with Q.Clear providing the best precision for small values of ${K}_{i}$ and small VOIs in all tissues.

**Conclusion:**

Q.Clear reconstruction of dynamic whole-body PET/CT data yields more precise ${K}_{i}$ values, especially for small values of ${K}_{i}$ and smaller VOIs, compared to standard OSEM. This precision improvement shows Q.Clear’s potential to better detect and characterize small lesion metabolic activity in oncology and allows for lower administered activity dosage.

## Introduction

Dynamic ^18^F-FDG PET/CT has emerged as a powerful tool for in-vivo glucose metabolic rate assessment in various tissues. This imaging technique is essential for understanding in-vivo metabolic processes and diagnosing a range of conditions, from cancer to metabolic disorders [1, 2]. The introduction of PET systems featuring high count rate sensitivity has enabled the development of dynamic whole-body PET acquisition protocols to assess ^18^F-FDG uptake rate (${K}_{i}$) using ^18^F-FDG PET/CT [3, 4]. With the introduction of these protocols, dynamic ^18^F-FDG PET/CT is no longer limited to a single bed position, which limits the axial field-of-view, but can be applied on a whole-body level. Dynamic PET/CT, however, faces several challenges, including potential patient motion, complex data analysis and increased examination time. In addition, as whole-body dynamic PET acquisitions, performed with short-axis field-of-view (SAFOV) PET/CT systems, require multiple bed positions to cover the whole-body range, the acquisition time per bed position for each dynamic time frame is strongly restricted. This results in higher image noise levels compared to dynamic single-bed and whole-body static PET/CT imaging. To compensate for this noise increase, higher administered ^18^F-FDG activity is typically needed to obtain sufficient image quality, which in turn leads to higher patient radiation dose [5].

In clinical practice, standard Ordered Subset Expectation Maximization (OSEM) algorithm [6] is the predominant method for PET image reconstruction [7, 8]. However, a major limitation of OSEM is the increase of image noise with an increasing number of iterations, which makes it necessary to stop the reconstruction iterations before full convergence has been reached to avoid excessive noise [8].

To address this limitation of OSEM, Bayesian penalized-likelihood reconstruction methods, e.g. the Block Sequential Regularized Expectation Maximization (BSREM) algorithm, have been developed. BSREM, like OSEM, is also an iterative reconstruction method but includes a noise-penalizing component that allows for more iterations, and therefore greater convergence at acceptable image noise levels. GE Healthcare’s implementation of BSREM, known as Q.Clear, controls the degree of noise penalization with a regularization parameter $\beta$ (8). Studies have demonstrated that the noise in Q.Clear reconstructed images is smaller than in OSEM reconstructed images [9, 10].

Despite the established benefits of Q.Clear in static imaging, its impact on pharmacokinetic modelling accuracy in dynamic ^18^F-FDG PET imaging has not been explored in detail. Ribeiro et al. investigated which $\beta$ value for the Q.Clear reconstruction gives the smallest bias with regards to the OSEM reconstruction for dynamic ^11^C-PHNO PET-MR scans [11]. In addition, based on a phantom study, Lysvik et al. determined the optimal value of $\beta$ for accurate quantification of dynamic ^18^F-PSMA-1007 PET scans, which was tested in two patients [12]. It is to be expected that this could be extrapolated to ^18^F-FDG PET imaging. However, to our knowledge, the impact of using a Bayesian penalized-likelihood PET reconstruction method on ${K}_{i}$, compared to standard OSEM, has not been investigated for dynamic whole-body ^18^F-FDG PET imaging using a SAFOV PET/CT system.

This study aims to investigate the potential improvements in both the bias and precision of ${K}_{i}$ in dynamic whole-body ^18^F-FDG PET/CT when utilizing Q.Clear compared to the standard-of-care OSEM method. To achieve a comprehensive assessment, a two-sided approach was used in which in-vivo data and computationally simulated time-activity curves were evaluated.

## Methods

### PET and image analysis

Data from two different data sets were used in this study.

The first dataset consisted of data from five overweight participants, obtained from a prior study in which dynamic whole-body ^18^F-FDG PET/CT scans were performed under hyperglycaemic and hyperinsulinemic conditions. During the hyperglycaemia, the glucose level in the participants was kept at 10 mmol/L. The participant characteristics are shown in Table 1.

**Table 1:** Participant characteristics of participants involved in the dynamic whole-body PET/CT research performed under hyperglycaemic and hyperinsulinemic conditions

Participant characteristics	Mean ± SD
Age (y)	67 ± 9
Weight (kg)	94.0 ± 15.8
BMI (kg/m^2^)	30.7 ± 3.4
Fasting glucose (mmol/L)	5.7 ± 0.3
Insulin fasted (mU/L)	9.5 ± 6.7
Insulin during PET (mU/L)	153.2 ± 32.7

This study was reviewed and approved by the Medical Ethics Review Committee of Maastricht University Medical Center and was registered as a clinical trial at clinicaltrials.gov with the identifier NCT04938544. In this study the participants received an activity dose of 3 MBq/kg.

The second dataset consisted of data from a 76-year-old female patient with lung cancer, obtained from an ongoing study in which dynamic whole-body ^18^F-FDG PET/CT scans are performed on lung cancer patients. This study was reviewed and approved by the Medical Ethics Review Committee of Maastricht University Medical Center and registered as a clinical trial at ClinicalTrials.gov under the identifier NCT05654675. In this study the participants received an activity dose of 2 MBq/kg.

**Fig. 1:**
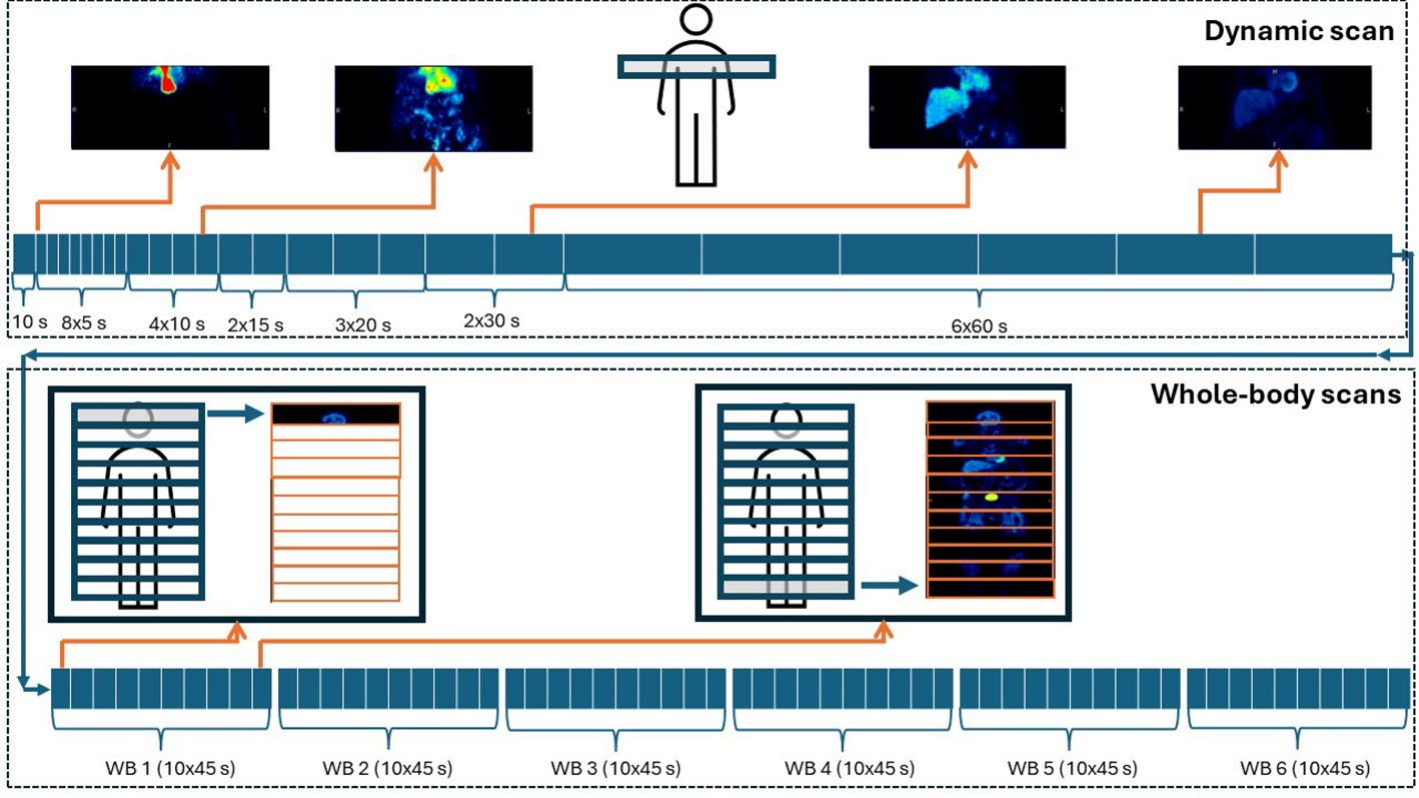
An illustration of the dynamic whole-body ^18^F-FDG PET/CT scanning protocol. The dynamic whole-body scan consisted of a 10 min dynamic scan of the thoracic region (frame durations of 1 × 10 s, 8 × 5 s, 4 × 10 s, 2 × 15 s, 3 × 20 s, 2 × 30 s and 6 × 60 s), which can be seen in the top part of the figure, followed by 6 whole-body scans of 10 bed positions each (45 s per bed position), which can be seen in the lower part of the figure. Between each whole-body scan there was a break of maximum 60 s

In both data sets, the PET/CT examination consisted of a low-dose CT-scan (120 kV, 30 mA, revolution time 0.5 s, pitch 0.98, and slice thickness 0.5 mm), followed by a 10 min single bed dynamic PET scan of the thoracic region (frame durations of 1 × 10 s, 8 × 5 s, 4 × 10 s, 2 × 15 s, 3 × 20 s, 2 × 30 s and 6 × 60 s) and 6 whole-body PET scans of 10 bed positions each (45 s per bed position) on the 5-ring GE Discovery MI PET/CT system. Between each whole-body scan there was a break of maximum 60 s. Figure 1 illustrates the dynamic whole-body PET scanning protocol.

The PET data were reconstructed using both the Q.Clear and the standard-of-care OSEM reconstruction method, which are both available on the GE Discovery MI PET/CT system. Reconstruction settings were chosen to meet the EARL 2 standards criteria [13]. For the Q.Clear reconstruction, a $\beta$ factor of 1200 was found to meet the EARL 2 standard. For the OSEM reconstruction, point spread function modelling (SharpIR) was turned on, the number of subsets equalled 17, the number of iterations equalled 3, the filter cutoff was set to 5 mm and the Z-axis filter was set to ‘heavy’ to comply with the EARL 2 standard. The images were reconstructed using a 256 × 256 matrix, with a voxel size of 2.73 mm × 2.73 mm × 2.80 mm. More information regarding the compliance of the images to the EARL 2 standard can be found in Appendix A.

**Fig. 2:**
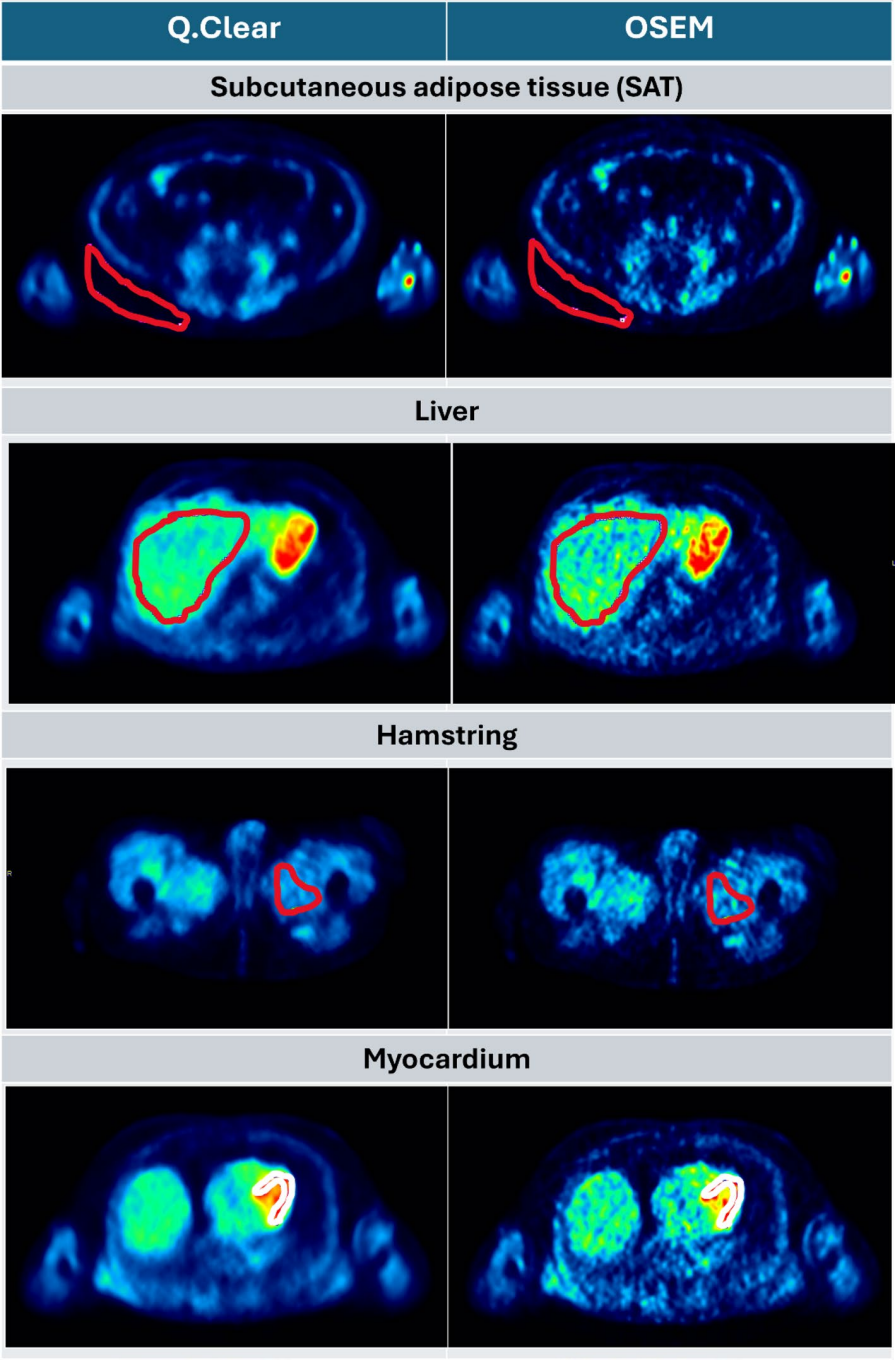
Example of the VOIs delineated of the SAT, the liver, the hamstring and the myocardium tissue on both the Q.Clear and the OSEM reconstructed images

In the first data set, VOIs of 4 tissue regions, subcutaneous adipose tissue (SAT), liver, hamstring, and myocardium, with apparent homogeneous ^18^F-FDG uptake were manually delineated in PMOD (Version 3.7, PMOD Technologies Ltd., Zurich, Switzerland) on the 6 whole-body PET frames which were reconstructed with the Q.Clear reconstruction method. The mean SAT VOI size was 134.2 $\pm$ 80.2 cm^3^, the mean liver VOI size was 559.6 $\pm$ 221.4 cm^3^, the mean hamstring VOI size was 81.4 $\pm$ 57.7 cm^3^ and the mean myocardium VOI size was 15.4 $\pm$ 9.5 cm^3^. These VOIs were used to obtain time activity curves (TACs) for the different tissues of the whole-body frames. Examples of the reconstructed images and the VOIs are shown in Fig. 2. It was chosen to analyse these tissues because they represent a large range of ${K}_{i}$ values, with SAT having a very low ${K}_{i}$ and the myocardium having a very high ${K}_{i}$ [1].

**Fig. 3:**
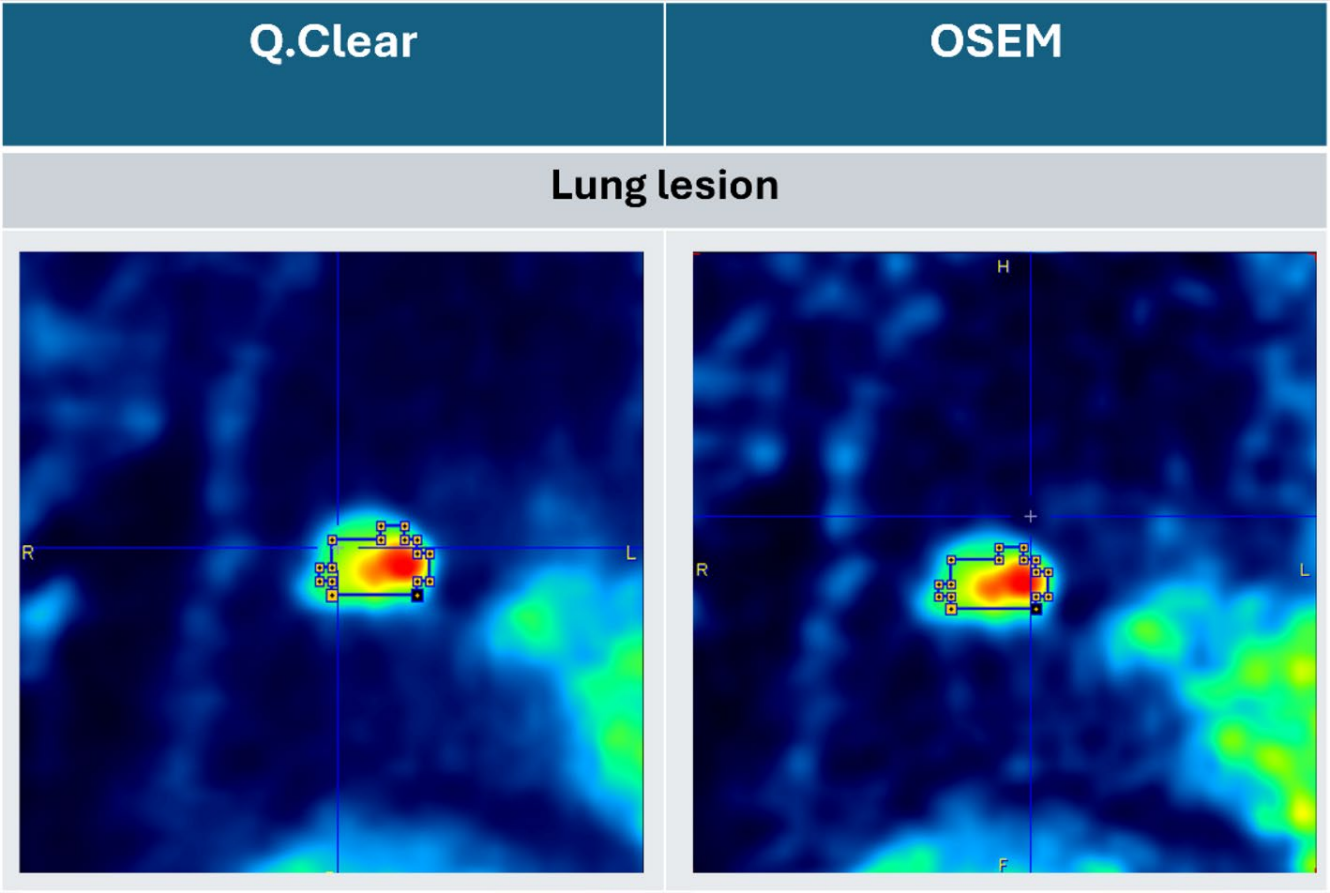
The VOI of the lung lesion delineated on both the Q.Clear and the OSEM reconstructed images

In the second data set, a VOI of a lung lesion was manually delineated in PMOD (Version 3.7, PMOD Technologies Ltd., Zurich, Switzerland) on the 6 whole-body PET frames which were reconstructed with the Q.Clear reconstruction method. This VOI was used to obtain the TAC for the lung lesion of the whole-body frames. The reconstructed images and the VOI are shown in Fig. 3. The size of this lesion VOI was 3.7 cm^3^.

The VOI of the descending aorta was manually delineated on the Q.Clear reconstructed dynamic scan and on the 6 whole-body frames. The delineated aorta volume was 3.2 $\pm$ 0.5 cm^3^. Using these VOIs, the arterial input function (AIF) was derived from the images.

The AIF was fitted to the following analytic function, as described by Vriens et al. [14]:

1 $$AIF(t) = \begin{cases}0 & t< \frac{a}{b}\\a \cdot t+b & -\frac{b}{a}\le t<\tau\\\sum_{i = 1}^{3} A_i \cdot e^{-\lambda_i (t-\tau)} & t \ge \tau   \end{cases}$$

The 8 free parameters in the fit ($a,b,{A}_{1},{A}_{2},{A}_{3},{\lambda }_{1},{\lambda }_{2} \text{and }{\lambda }_{3}$) were determined using a combination of a linear fit ($t<\tau$) and a nonlinear least squares fit ($t\ge \tau$). $\tau$ is the time after ^18^F-FDG injection until the highest activity concentration in the descending aorta has been reached, and is defined as the time of the dynamic frame with the highest activity concentration in the aorta. The values of the free parameters for both reconstruction methods are given in Appendix B.

### VOI voxel noise determination

The voxel noise in all tissue VOIs was obtained by means of subsequent frame subtraction to eliminate potential spatial inhomogeneities in radiotracer uptake. This approach was preferred over calculating the standard deviation of voxel values within the VOIs, as the latter not only captures the noise but also incorporates variability due to the heterogeneity of radiotracer uptake. As a result, the standard deviation is likely to overestimate the true noise present within the VOI. In contrast, frame subtraction eliminates heterogeneous radiotracer uptake to a large extent and thus allows for a more accurate measurement of image noise.

In the calculation of the voxel noise, a Poisson noise distribution is assumed. To calculate the voxel noise in the tissue VOIs, subsequent frames were scaled such that the mean pixel value in a particular VOI was the same in both frames. This was done by multiplying the voxel values in the former frame by the factor ${c}_{1}=\frac{{\mu }_{m}}{{\mu }_{1}}$ and the voxel values in the latter frame by the factor ${c}_{2}=\frac{{\mu }_{m}}{{\mu }_{2}}$, where ${\mu }_{1}$ and ${\mu }_{2}$ are the mean voxel values of the VOI in the former and latter frame, respectively, and ${\mu }_{m}$ is the average value of ${\mu }_{1}$ and ${\mu }_{2}$. Then the scaled frames were subtracted from each other and the standard deviation ${\sigma }_{sub}$ of the voxel values in the VOI from the subtracted frame was determined. This standard deviation was then multiplied by a frame specific constant to obtain the tissue VOI voxel noise ${\sigma }_{tissue}$ in both frames:

2 $$\sigma_{tissue} = \frac{{\sigma_{sub} }}{{\sqrt { c_{1}^{2} + c_{2}^{2} } }}$$

In the case that a frame was adjacent to two frames (e.g. frame 2, which is adjacent to both frame 1 and frame 3), the voxel noise was defined as the average of the values calculated using the frame before and the frame after. This process was repeated for all tissue VOIs and all whole-body frames. The noise ${\sigma }_{tissue}$ is the reported absolute noise level.

The standard error in the mean voxel value ${\nu }_{\mu ,tissue}$ in a tissue VOI is defined by:

3 $$\nu_{\mu ,tissue} = \frac{{\sigma_{tissue} }}{{\sqrt {N_{vox,tissue} } }}$$

where ${N}_{vox,tissue}$ is the number of voxels in the tissue VOI.

The voxel noise in the descending aorta region (${\sigma }_{aorta})$ was defined as the standard deviation of the voxel values in that region on each of the dynamic and whole body frames.

Similar to the VOIs, the standard error in the mean value of the aorta region ${\nu }_{\mu ,aorta}$ is defined by:

4 $$\nu_{\mu ,aorta} = \frac{{\sigma_{aorta} }}{{\sqrt {N_{vox,aorta} } }}$$

where ${N}_{vox,aorta}$ is the number of voxels in the delineated aorta region.

### In-vivo measurements

The impact of the two reconstruction methods on the value and precision of the ^18^F-FDG influx rate, ${K}_{i}$, in the in-vivo participant data was investigated using a bootstrapping resampling method [15]. Here the measured AIF and TACs of the SAT, liver, hamstring, myocardium and lung lesion VOIs from the Q.Clear and the OSEM reconstructed images of each participant were used as input parameters, along with the standard errors of the means of the tissue (${\nu }_{\mu ,tissue}$) and aorta (${\nu }_{\mu ,aorta}$) regions, calculated with Eqs. 3 and 4, respectively. Bootstrapping was performed using a Monte Carlo (MC) simulation in which noise was added to the TACs and AIF in each run of the simulation. A total of 1000 MC runs were performed per simulation. The noise added was randomly sampled from a Gaussian distribution with zero mean and standard deviation ${\nu }_{\mu ,tissue,Q.Clear}$ and ${\nu }_{\mu ,tissue,OSEM}$ for the Q.Clear and the OSEM reconstructed images, respectively. For the AIF, noise was added in a similar fashion, using a Gaussian distribution with zero mean and standard deviation ${\nu }_{\mu ,aorta, Q.Clear}$ and ${\nu }_{\mu ,aorta,OSEM}$ for the Q.Clear and the OSEM reconstructed images, respectively.

For each MC run, the ${K}_{i}$ value of a particular VOI was calculated using graphical Patlak analysis [16]. The mean ${K}_{i}$ value of the MC simulation was determined and the precision of ${K}_{i}$ was defined as the standard deviation in ${K}_{i}$ across all MC runs.

For each tissue type, the MC simulations were repeated using standard errors of the means corresponding to VOI sizes of 27 voxels (i.e. 0.56 cm^3^) to assess the effect of smaller VOI sizes on the calculated ${K}_{i}$ values.

### Artificial TAC generation for ^18^F-FDG influx rate evaluation

To investigate the impact of the two reconstruction methods on the bias of ${K}_{i}$, artificial TACs of regions with a set ^18^F-FDG influx rate ${K}_{i,true}$ were generated. The method of the generation of the artificial TACs using the 2 tissue compartment model is described in Appendix C. The values of ${K}_{i,true}$ and the corresponding rate constants ${K}_{1}$, ${k}_{2}$ and ${k}_{3}$ were established by fitting the 2 tissue compartment model to the TACs of the SAT, liver, triceps and myocardium VOI of one participant. The TACs were determined over the full scan length, so on both the 10-min dynamic scan of the thoracic region and the 6 whole body passes. The triceps was chosen over the hamstring because the triceps are contained in the 10-min dynamic scan of the thoracic region, while the hamstrings are not. The determined values of ${K}_{i,true}$ and the rate constants are shown in Table 2.

**Table 2:** The values of ${K}_{i,true}$ (unit = $\cdot$ 10^–3^ ml $\cdot$ cm^−3^ $\cdot$ min^−1^), ${K}_{1}$ (unit = ml·min^−1^·g^−1^), ${k}_{2}$ (unit =  min^−1^) and ${k}_{3}$ (unit = min^−1^) for the SAT, liver, triceps and myocardium determined for one participant using the 2 tissue compartment model

Tissue	${K}_{i,true}$	${K}_{1}$	${k}_{2}$	${k}_{3}$
SAT	0.37	0.0032	0.083	0.011
Liver	12.4	0.36	0.45	0.016
Triceps	13.8	0.025	0.22	0.27
Myocardium	46.2	2.74	4.84	0.083

Per reconstruction method, the AIF of one study participant was used to generate four different TACs, where each TAC corresponded to a different value of ${K}_{i,true}$ as shown in Table 2. The AIF of one study participant was chosen such that a realistic AIF was used to generate the TACs. Once the TACs had been generated, a bootstrapping resampling method was performed in which noise was randomly added to the AIF and the TACs during each run of the MC simulation as described in Sect. “In-vivo measurements”. The tissue VOI noise ${\nu }_{tissue}$ was taken as a percentage of the ^18^F-FDG concentration. This noise percentage was determined from the noise present in the VOIs in the in-vivo images of the participants in the tissues shown in Table 2, for both types of reconstruction. For the triceps, the noise percentage from the hamstrings in the in-vivo images was used. From the generated TACs and corresponding AIF, the ^18^F-FDG influx rate was again calculated using graphical Patlak analysis.

The number of voxels present in the VOI was set to 1000 voxels (20.7 cm^3^) and 27 voxels (0.56 cm^3^) to determine the effect of VOI size on the bias and precision of both reconstruction methods. The VOI size of 1000 voxels was chosen because it is representative of the size of an in-vivo VOI and the VOI size of 27 voxels was chosen because it is representative of a small VOI containing 3 × 3 × 3 voxels. The number of voxels in the aorta region was set to the average in vivo VOI size of 153 voxels (3.19 cm^3^).

The calculated ^18^F-FDG influx rate (${K}_{i,calc}$) was defined as the mean ^18^F-FDG influx rate during the simulations and compared to the true ^18^F-FDG influx rate (${K}_{i,true}$) to determine the bias of ${K}_{i,calc}$. The bias was defined as ${K}_{i,calc}-{K}_{i,true}$ and was also expressed as a percentage of ${K}_{i,true}$. The precision of ${K}_{i,calc}$ was calculated as the standard deviation of the ^18^F-FDG influx rates across all MC simulation runs (${\sigma }_{{K}_{i,calc}}$) and was also expressed as a percentage of ${K}_{i,true}$.

### Statistical analysis

The amount of VOI voxel noise was compared between Q.Clear and OSEM using a parametric one-sided paired *t*-test with a significance level of 0.05, and the ^18^F-FDG influx rate precision in the in-vivo measurements was compared between Q.Clear and OSEM using a one-sided Wilcoxon signed-rank test, with a significance level of 0.05. Bland Altman plots were used to determine the difference in the ^18^F-FDG influx rate calculated with both reconstruction methods in the in-vivo measurements and to determine the bias of the ^18^F-FDG influx rate calculated with both reconstruction methods (${K}_{i,calc}$) with regards to the gold-standard (${K}_{i,true}$).

## Results

### Noise level

In the liver, the mean voxel noise over the six whole-body frames over all participants, quantified by the standard deviation of voxel values, was 0.48 kBq/ml and 1.08 kBq/ml for Q.Clear and OSEM reconstructed frames, respectively. When using frame subtraction, the mean voxel noise measured was 0.42 kBq/ml and 1.12 kBq/ml, respectively.

The voxel noise determined using frame subtraction was found to be highly comparable to the voxel noise determined by voxel standard deviation, justifying the use of the frame subtraction method for the other VOIs.

**Fig. 4:**
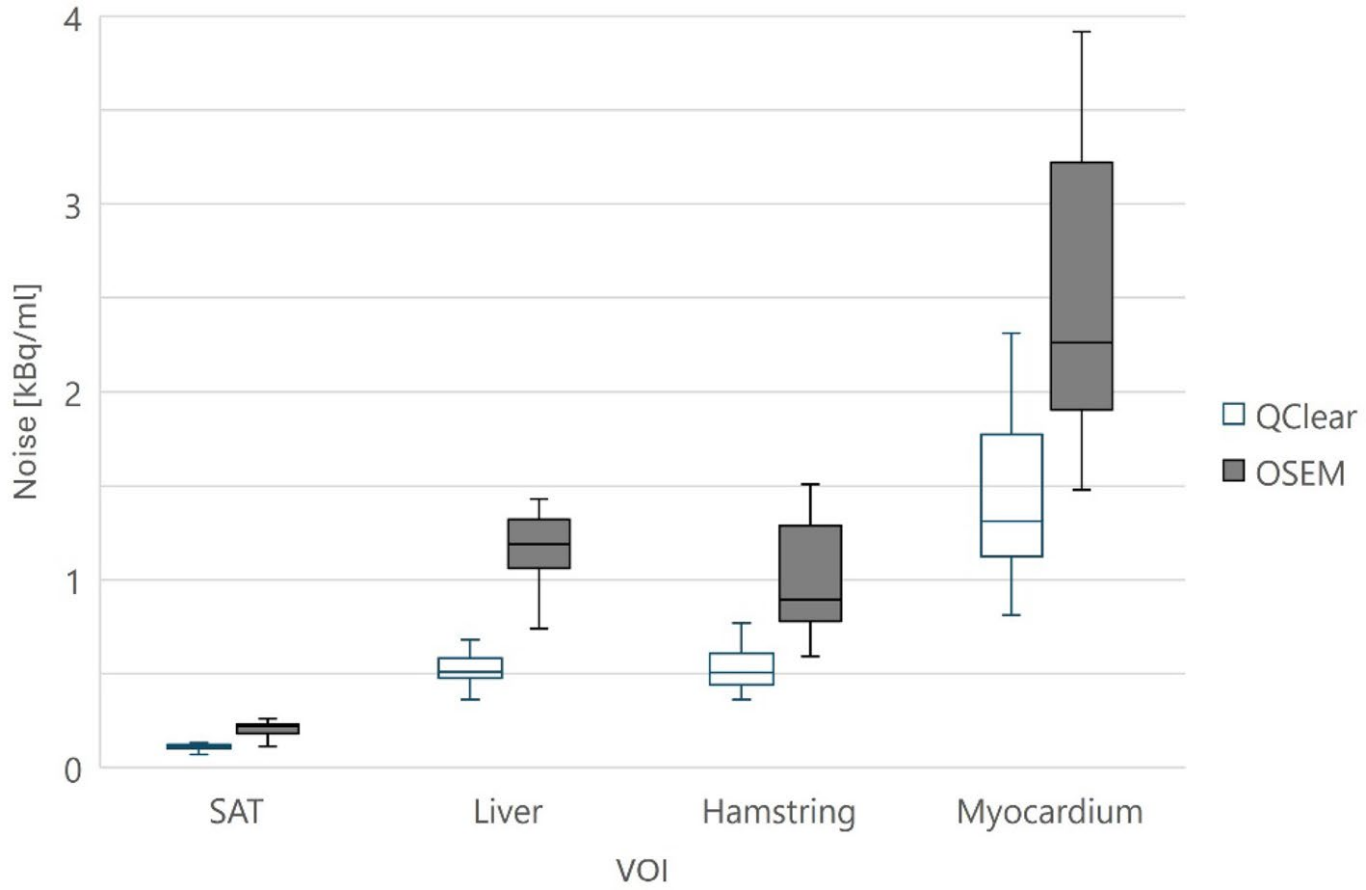
The absolute noise levels in the SAT, liver, hamstring and myocardium VOIs over all participants in the six Q.Clear and OSEM reconstructed whole-body frames. The length of the whiskers in the box-plot are equal to 1.5 times the interquartile range. In all VOIs the mean noise is lower in the Q.Clear reconstructed images compared to the OSEM reconstructed images (*p* < 0.05)

Figure 4 presents the absolute voxel noise levels in the VOIs of the SAT, liver, hamstring and myocardium across all participants in the six whole-body frames. The mean voxel noise in all VOIs was 40–55% lower in the Q.Clear compared to the OSEM reconstructed images, which was a significant difference (*p* < 0.05).

The mean relative voxel noise levels in the SAT, liver, hamstring and myocardium VOIs across all participants are listed in Table 3. It was found that the relative noise level, defined as the noise relative to the mean voxel value in a VOI, is consistently lower in all tissues for the Q.Clear compared to the OSEM reconstructed images. The relative noise level was highest in the SAT and is lowest in the hamstring and myocardium. These relative voxel noise levels were used in the simulations with the simulated TACs, as described in Sect. “Artificial TAC generation for 18F-FDG influx rate evaluation”, for the ${K}_{i,true}$ values of 0.37, 12.4, 13.8 and 46.2 $\cdot$ 10^–3^ ml $\cdot$ cm^−3^ $\cdot$ min^−1^, respectively.

**Table 3:** The mean relative voxel noise across all participants in regions with different values of ${K}_{i,true}$ (unit: $\cdot$ 10^–3^ ml $\cdot$ cm^−3^ $\cdot$ min^−1^), for the Q.Clear and OSEM reconstruction method

Tissue	${K}_{i,true}$	Relative noise level	
		Q.Clear (%)	OSEM (%)
SAT	0.37	22	39
Liver	12.4	11	24
Hamstring	13.8	9	16
Myocardium	46.2	9	16

### In-vivo measurements

In Table 4, the median and range of the precision of the FDG influx rate ${K}_{i}$ across the participants is given for both the full and small sized SAT, liver, hamstring and myocardium VOIs, when calculated with the Q.Clear and the OSEM reconstructed images for the in-vivo measurements. Furthermore, the average value of the ^18^F-FDG influx rate across all participants calculated with the Q.Clear reconstructed images is provided for each tissue type. For one participant, it was not possible to calculate ${K}_{i}$ of the SAT due to too much movement of the participant during the scan. The results for the SAT are thus based on the measurements from four participants instead of five. For the lung lesion VOI, the ^18^F-FDG influx rate and the precision of the ^18^F-FDG influx rate of the single lung lesion participant are also shown in Table 4. Because this concerns only one participant, no p-value is given.

**Table 4:** Mean value of the ^18^F-FDG influx rate ${K}_{i}$ (unit: $\cdot$ 10^–3^ ml $\cdot$ cm^−3^ $\cdot$ min^−1^) across all participants calculated using the Q.Clear reconstructed images and the corresponding precision in ${K}_{i}$ (median + range across the participants) which was calculated using both the Q.Clear and the OSEM reconstructed images for the full VOI size and the reduced VOI size of 27 voxels. The median value of the precision is also given as a percentage of the mean ^18^F-FDG influx rate. In the final column the *p*-values from the Wilcoxon signed-rank test are given, indicating the statistical significance of the difference in precision between the reconstruction methods. For the lung lesion VOI, the 18F-FDG influx rate and the precision of the ^18^F-FDG influx rate of the single lung lesion participant are shown in the table. Because this concerns only one participant, no *p*-value is given.

VOI size	Tissue	Average ${K}_{i}$	Precision in ${K}_{i}$		p-value per VOI
			Q.Clear	OSEM	
Full size	SAT	1.4	0.050 (3.6%) [0.021–0.091]	0.050 (3.6%) [0.026–0.060]	0.36
	Liver	6.7	0.69 (10.3%) [0.086–0.98]	0.46 (6.9%) [0.070–1.1]	0.45
	Hamstring	23.2	0.18 (0.8%) [0.062–0.39]	0.19 (0.8%) [0.087–0.31]	0.25
	Myocardium	49.8	0.54 (1.1%) [0.18–0.86]	0.70 (1.4%) [0.24–4.8]	0.034
	Lung lesion	21.4	0.34 (1.6%)	0.35 (1.6%)	-
27 voxels	SAT	1.4	0.10 (7.1%) [0.097–0.11]	0.16 (11.4%) [0.15–0.17]	0.034
	Liver	6.7	0.61 (9.1%) [0.31–1.2]	0.93 (13.9%) [0.59–1.3]	0.040
	Hamstring	23.2	0.42 (1.8%) [0.29–0.55]	0.69 (3.0%) [0.60–1.0]	0.022
	Myocardium	49.8	1.3 (1.1%) [0.95–2.2]	2.1 (4.2%) [1.3–14]	0.022
	Lung lesion	21.4	0.61 (2.9%)	0.77 (3.6%)	-

For the full VOI sizes, the use of Q.Clear leads to a significantly better precision in ${K}_{i}$ only for the myocardium (*p* < 0.05). For the SAT, liver and hamstring VOI, the *p*-value ranged between 0.25 and 0.45, indicating no significant difference. However, for the small sized VOIs, the precision in ${K}_{i}$ is significantly better when calculated using the Q.Clear compared to the OSEM reconstructed images in all tissue regions (*p* < 0.05).

**Fig. 5:**
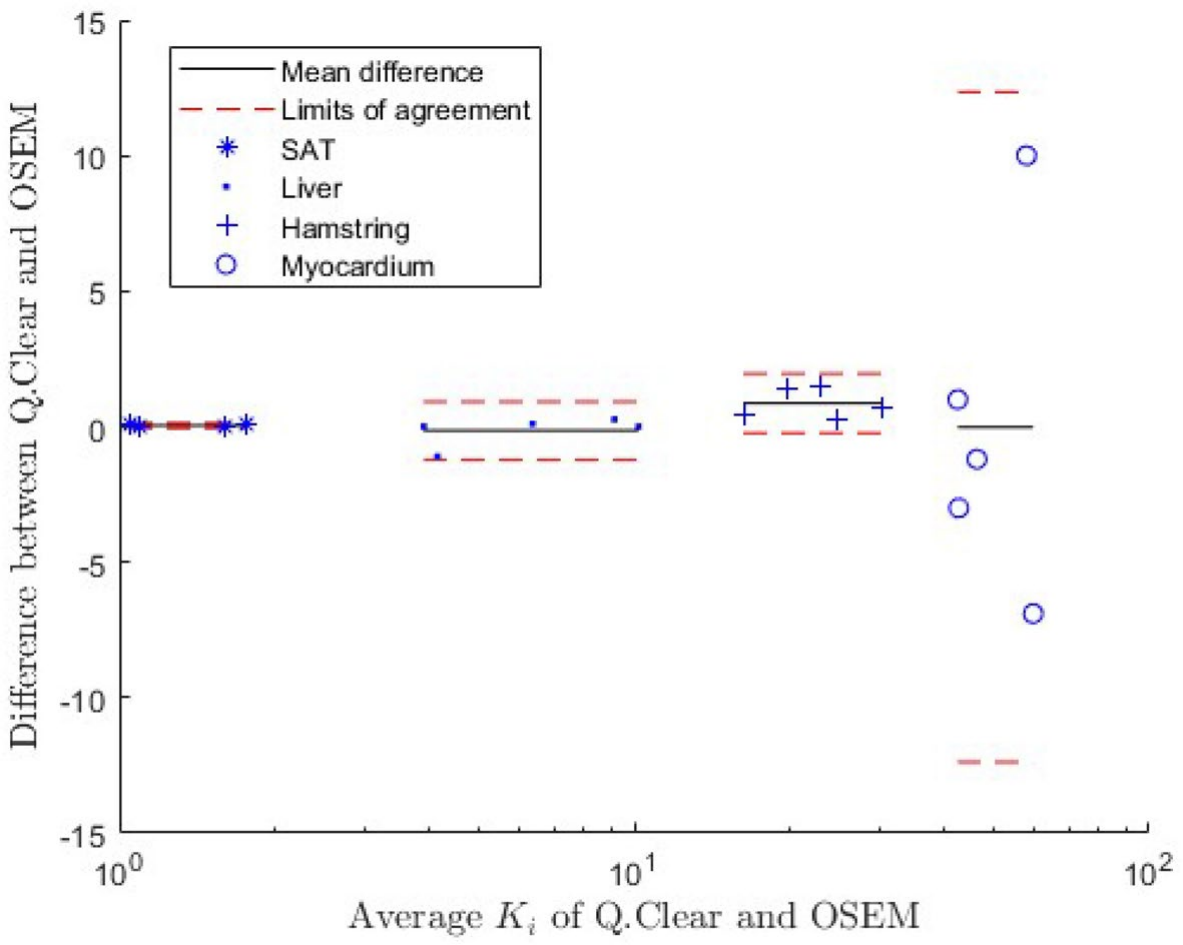
Bland–Altman plot showing the difference in ^18^F-FDG influx rate assessed on the Q.Clear and the OSEM reconstructed images for the full VOI size. The units of ${K}_{i}$ are $\cdot$ 10^–3^ ml $\cdot$ cm^−3^ $\cdot$ min^−1^, both on the x-axis and y-axis of the plot. The mean difference and the limits of agreement are shown separately for each tissue

The difference in ^18^F-FDG influx rate between the two reconstruction methods is depicted in a Bland–Altman plot in Fig. 5 for the full-sized SAT, liver, hamstring and myocardium VOIs. For these full sized VOIs, the mean difference of ${K}_{i}$ calculated based on Q.Clear compared to OSEM reconstructed images is 0.19 $\cdot$ 10^–3^ ml $\cdot$ cm^−3^ $\cdot$ min^−1^, with the relative difference per region between 0.04% for the myocardium and 3.7% for the hamstring. For the reduced size SAT, liver, hamstring and myocardium VOIs of 27 voxels the mean difference equals 0.13 $\cdot$ 10^–3^ ml $\cdot$ cm^−3^ $\cdot$ min^−1^, with the relative difference per region between 0.3% for the myocardium and 3.6% for the hamstring. The difference for both the full and reduced VOI size is in the same range as the precision obtained with both reconstruction techniques, as shown in Table 4. This indicates that the difference between the reconstruction methods is small.

For the lung lesion VOI, the difference in ^18^F-FDG influx rate between the two reconstruction methods is 1.3 $\cdot$ 10^–3^ ml $\cdot$ cm^−3^ $\cdot$ min^−1^ for the full-sized VOI and 1.4 $\cdot$ 10^–3^ ml $\cdot$ cm^−3^ $\cdot$ min^−1^ for the reduced size VOI.

### Simulations with artificial TACs

The precision in ${K}_{i,calc}$ for both reconstruction methods is shown in Table 5 for different values of ${K}_{i,true}$ and the two VOI sizes. The results in the table indicate that the precision in ${K}_{i,calc}$ using Q.Clear is better than or similar to OSEM for the VOI size of 1000 voxels. For the small VOI size, however, Q.Clear systematically outperforms OSEM in terms of ${K}_{i}$ precision, showing the largest relative difference in precision between both methods for small ^18^F-FDG influx rates (0.37 and 12.4 $\cdot$ 10^–3^ ml $\cdot$ cm^−3^ $\cdot$ min^−1^). The relative bias between ${K}_{i,calc}$ with noise added to the simulations compared to ${K}_{i,true}$ without noise was small for both reconstruction methods: for Q.Clear the maximum bias was 0.8% and for OSEM the maximum bias was 0.9%.

**Table 5:** The precision of ${K}_{i,calc}$ (unit = $\cdot$ 10^–3^ ml $\cdot$ cm^−3^ $\cdot$ min^−1^) for a range of set FDG influx rate values (${K}_{i,true}$) and VOI sizes of 1000 voxels and 27 voxels, based on both the Q.Clear and OSEM reconstruction. The precision is also given as a percentage of ${K}_{i,true}$

VOI size (#voxels)	${K}_{i,true}$	Precision in ${K}_{i}$	
		Q.Clear	OSEM
1000	0.37	0.00056 (1.5%)	0.00074 (2.0%)
	12.4	0.12 (0.98%)	0.17 (1.4%)
	13.8	0.062 (0.46%)	0.080 (0.58%)
	46.2	0.19 (0.41%)	0.27 (0.58%)
27	0.37	0.024 (6.7%)	0.045 (12.4%)
	12.4	0.33 (2.7%)	0.72 (5.8%)
	13.8	0.25 (1.9%)	0.43 (3.1%)
	46.2	0.83 (1.8%)	1.6 (3.5%)

## Discussion

This study investigated the impact of the use of the Q.Clear versus the OSEM reconstruction method on the bias and precision of the net ^18^F-FDG influx rate calculation in dynamic whole-body PET/CT imaging using SAFOV PET systems. To the best of our knowledge, this study is the first to directly compare the bias and precision of the quantitative ^18^F-FDG influx rate between Q.Clear and OSEM.

### Noise

Overall, Q.Clear images exhibited significantly lower noise levels across all tissue VOIs compared to OSEM, consistent with previous studies which showed lower noise in both in-vivo and phantom studies [9, 18]. The excellent agreement between noise levels determined by frame subtraction and the standard deviation of liver VOI voxel values confirms the suitability of frame subtraction for VOI noise assessment.

### Precision

For the reduced VOI size of 27 voxels in the in-vivo measurements, the precision of the ^18^F-FDG influx rate based on Q.Clear was significantly better compared to OSEM for the SAT, liver, hamstring and myocardium. In contrast, for the full-sized VOIs, a statistically significant difference in ${K}_{i}$ precision between both reconstruction methods was only observed for the myocardium which has a relatively small VOI size compared to the other tissue types. This observation is in line with expectations, as the reduced VOI size increases the effect of the noise on the variation of the TACs in the Patlak analysis. The difference in noise levels between the Q.Clear and the OSEM images has, therefore, a clear impact on the precision of ${K}_{i}$. Furthermore, the difference in relative ${K}_{i}$ precision between Q.Clear and OSEM is largest for the SAT and the liver, showing a relatively low ^18^F-FDG influx rate. This suggests that the use of Q.Clear in dynamic whole-body PET/CT is particularly beneficial for ${K}_{i}$ calculations of small regions exhibiting a low ^18^F-FDG influx rate. This finding is supported by the artificial TAC simulations, showing that the ${K}_{i}$ precision based on the Q.Clear reconstructed images is consistently lower compared to the OSEM reconstructed images for the small VOI size of 27 voxels.

For the lung lesion VOI, the ${K}_{i}$ precision based on Q.Clear was also better compared to OSEM, for both the full-sized VOI and the reduced size VOI. However, further research including more participant data is needed to show the statistical significance of this difference for lesions. Even though the difference in ${K}_{i}$ precision between Q.Clear and OSEM for the full-sized VOIs was shown to be significant for the myocardium, the actual relative difference of the median precision values between the reconstructions was only 0.3%, which is very small compared to the precision differences found in the small VOI sizes. The precision improvement with Q.Clear could be beneficial for the practical implementation of dynamic ^18^F-FDG PET/CT scanning. Firstly, the use of Q.Clear could potentially lead to better detection and characterization of small regions using dynamic whole-body PET/CT imaging. This could for instance be of great value for the detection and characterization of small tumors, metastases, lymph nodes and inflammation, warranting further research and confirmation. Alternatively, the precision improvement could also leave room to reduce the administered ^18^F-FDG activity or to shorten the acquisition time of the dynamic whole-body ^18^F-FDG PET/CT scan, while preserving the ^18^F-FDG influx rate precision that is obtained when using OSEM. Reduction of the administered FDG activity would reduce the patient radiation dose and potentially open up new possibilities for longitudinal dynamic whole-body PET/CT study protocols of in particular healthy volunteers receiving one (or multiple) follow-up scans that are nowadays sometimes limited by the radiation exposure of the volunteer. Conversely, shortening the scan time would increase patient comfort and make dynamic scanning more feasible in the clinic.

### Bias

The small difference in the mean ^18^F-FDG influx rate for the in-vivo patient data for the Q.Clear and OSEM reconstructed images suggests a similar bias for both reconstruction methods. Moreover, the magnitude of this difference lies within the range of the precision of both methods, indicating that the difference between the reconstruction methods is not substantial. This is in line with expectations, as the AIF and TACs of the VOIs are expected to be similar for both reconstruction methods because the reconstruction methods conform to the EARL 2 standard, leading to similar recovery coefficients and therefore similar mean voxel values in the VOIs.

It is noteworthy that the difference in the ^18^F-FDG influx rate for the myocardium VOI calculated using both methods is relatively large compared to the other VOIs. For each participant, the same myocardium VOI was used on both the Q.Clear and OSEM reconstructed images and the VOI was adjusted to each time frame to compensate for movement between the whole-body passes. The VOIs were determined on the Q.Clear reconstructed PET images. On these images, defining the boundary of the myocardium based solely on ^18^F-FDG uptake can be challenging, potentially leading to incorrect delineation of part of the myocardium. The myocardium VOI is also relatively small compared to the other VOIs of the other tissues, leading to a larger uncertainty in the average voxel values in the TAC. In addition, regular contraction of the myocardium leads to movement during and between each frame, which can result in a mismatch of the myocardium with the delineated VOI. The mismatch between the myocardium and VOI has a relatively large effect on the mean voxel value compared to the other tissues due to the partial volume effect, which is influenced by the shape of the myocardium. The myocardium has a relatively large surface area compared to the volume, leading to a significant portion of the myocardium falling outside the VOI in the case of movement. The high contrast in ^18^F-FDG uptake between the myocardium and surrounding tissue exacerbates the partial volume effect.

From the simulations with the artificial TACs, the absence of a systematic significant bias for both Q.Clear and OSEM indicates that the reconstruction methods do not inherently skew the ^18^F-FDG influx rate and are both reliable for determining the influx rate. Furthermore, the bias is not dependent on the size of the ^18^F-FDG influx rate and there is no direct relationship between the bias and the magnitude of the noise, which increases for increasing ^18^F-FDG influx rate. These findings are insightful as they highlight that while noise levels vary with influx rate, this variability does not translate into a varying bias in the influx rate. These results show that with regards to bias, both reconstruction methods provide reliable results with respect to the gold standard ^18^F-FDG influx rate.

### Difference between in-vivo measurements and simulations with artificial TACs

The in-vivo patient measurements and the simulations with the artificial TACs were both used to determine the precision of the ^18^F-FDG influx rate for VOIs with different metabolic characteristics. It was chosen to use both methods because the precision results of the in-vivo measurements warranted confirmation from simulations with artificial TACs and the creation of artificial TACs allowed for the calculation of the bias of the reconstruction methods with regards to the gold standard ^18^F-FDG influx rate.

While the computer simulations provide a controlled environment to assess the precision and potential biases up to a certain extent, they do not take into account the exact same conditions as the in-vivo measurements. For instance, in the simulations an artificial TAC is created to which noise is added. The artificial TAC however does not take into account effects that may affect the real-life measurement of a TAC, like partial volume effects and physiological motion, such as respiratory or cardiac movement. This could explain why in some cases there is a relatively large difference between the size of the precision obtained with the in-vivo patient data versus the simulations with the artificial TACs. Despite this discrepancy, the simulations with the artificial TACS do provide a useful framework for understanding the relationship between the reconstruction methods and the precision of the ^18^F-FDG influx rates. Most importantly, both the in-vivo measurements and the simulations with the artificial TACs show that the relative difference in precision between Q.Clear and OSEM is largest for small VOIs and for tissues with small ^18^F-FDG influx rates of approximately 0.37 and 12.4 $\cdot$ 10^–3^ ml $\cdot$ cm^−3^ $\cdot$ min^−1^, with Q.Clear providing the best precision.

### Practical implications of Q.Clear and OSEM

While Q.Clear provides reconstructed images with less noise due to its Bayesian penalized-likelihood approach, there are several important practical differences between Q.Clear and OSEM which should be taken into account in their use. Firstly, OSEM is faster and computationally less demanding than Q.Clear [10]. OSEM can therefore be more beneficial in clinical settings where rapid turnaround times are essential or when the computational resources are limited. The increased computational demand might be a limiting factor for Q.Clear, in particular in dynamic whole-body PET/CT applications where images need to be reconstructed for many different time frames with short durations. However, the benefits in image quantification and the resulting diagnostic accuracy could justify its use.

Secondly, both Q.Clear and OSEM require parameter adjustments to optimize image quality. OSEM requires the fine-tuning of the number of iterations and subsets and the image smoothing filter. This gives the user a lot of flexibility to adjust settings based on the specific image requirements, but the optimization of the reconstruction parameters could be a complex procedure. In contrast. Q.Clear only requires the adjustment of the penalizing factor ($\beta$). This simplifies the optimization process in finding the right reconstruction parameters.

Finally, the availability of Q.Clear could be a limiting factor in its widespread implementation in dynamic whole-body ^18^F-FDG PET/CT imaging. Q.Clear is a proprietary reconstruction algorithm developed by GE Healthcare, meaning that hospitals using PET/CT systems from other manufacturers do not yet have access to Q.Clear. In contrast, OSEM is more widely available and is supported by a wide range of PET/CT systems from various manufacturers. This broader availability makes OSEM a more accessible option for many hospitals.

### Study limitations

Several study limitations should be noted for this study. Firstly, the study population for the in-vivo measurements was small and may increase the uncertainties in the results. While the findings of this study provide valuable insights into the precision in ^18^F-FDG influx rate calculations using Q.Clear reconstructed images, the limitation of a small sample size should be considered when interpreting the results. Secondly, the study participants of one of the data sets were scanned under hyperinsulinemic and hyperglycaemic conditions, which could lead to different ^18^F-FDG influx rates of the tissues than under normal conditions, i.e. when a patient is in a fasted state. This is also seen in our results when comparing them to literature. E.g. Liu et al. found that the ^18^F-FDG influx rates of liver, myocardium and muscle tissue are 10.1, 42.2 and 3.3 ml $\cdot$ cm^−3^ $\cdot$ min^−1^, respectively, under normal conditions [17], which are all lower than the ^18^F-FDG influx rates found in these tissues in our in-vivo measurements. Despite the fact that the ^18^F-FDG uptake rates found in our study may be different from those found under normoglycemia and normal insulin concentrations, our results show that Q.Clear outperforms OSEM with regards to precision across the whole range of ^18^F-FDG uptake rates. It is reasonable to assume that this advantage holds true under normal conditions as well. Finally, the study’s in-vivo measurements were mainly conducted solely on relatively healthy individuals, while only one participant with a lung lesion was included. This limitation means we did not extensively evaluate the difference in ^18^F-FDG influx rate precision between Q.Clear and OSEM in various pathological conditions, such as small tumors, metastases, lymph nodes, and inflammation. However, the included participant with a lung lesion did show an improved ^18^F-FDG uptake rate precision for both the full sized VOI and the reduced size VOI. Furthermore, Q.Clear consistently provides superior precision in smaller volumes, which may serve as a proxy for small pathological structures like tumors and metastases. Such small structures tend to exhibit elevated noise levels compared to larger volumes due to reduced number of total counts present in the VOI, a characteristic also seen in the small volumes assessed in this study. Thus, Q.Clear is likely advantageous for the metabolic assessment of these conditions, even though they were not extensively analyzed in this study. The enhanced precision with Q.Clear could be crucial for early detection and monitoring. Future research involving diverse patient populations will be essential to confirm the broader applicability of Q.Clear in these contexts.

Finally, another limitation of this study is that it primarily provides a conceptual demonstration rather than direct evidence of clinical utility. Although our findings highlight reduced noise levels and improved precision in ^18^F-FDG influx rate calculations with Q.Clear, their practical implications in clinical settings remain unexplored. Specifically, we did not assess whether these improvements influence lesion detection rates, diagnostic accuracy, or treatment decision-making. Additionally, the study does not offer recommendations regarding potential reductions in dose or scan time based on these results. Addressing these questions would require dedicated investigations into the clinical outcomes associated with using Q.Clear. Future studies should aim to bridge this gap to better define the method’s relevance to clinical practice.

## Conclusion

This study showed that Q.Clear consistently provides lower noise levels and improved precision in ^18^F-FDG influx rate calculations in dynamic whole-body PET/CT scans, particularly for small VOIs with low influx rates. This advantage is evident in both in-vivo patient measurements and simulations with artificial TACs, suggesting its potential for optimizing dynamic whole-body PET/CT imaging, particularly in clinical applications requiring detailed metabolic assessments of small lesion volumes or low influx rate values.

## Figures and Tables

**Fig. 6:**
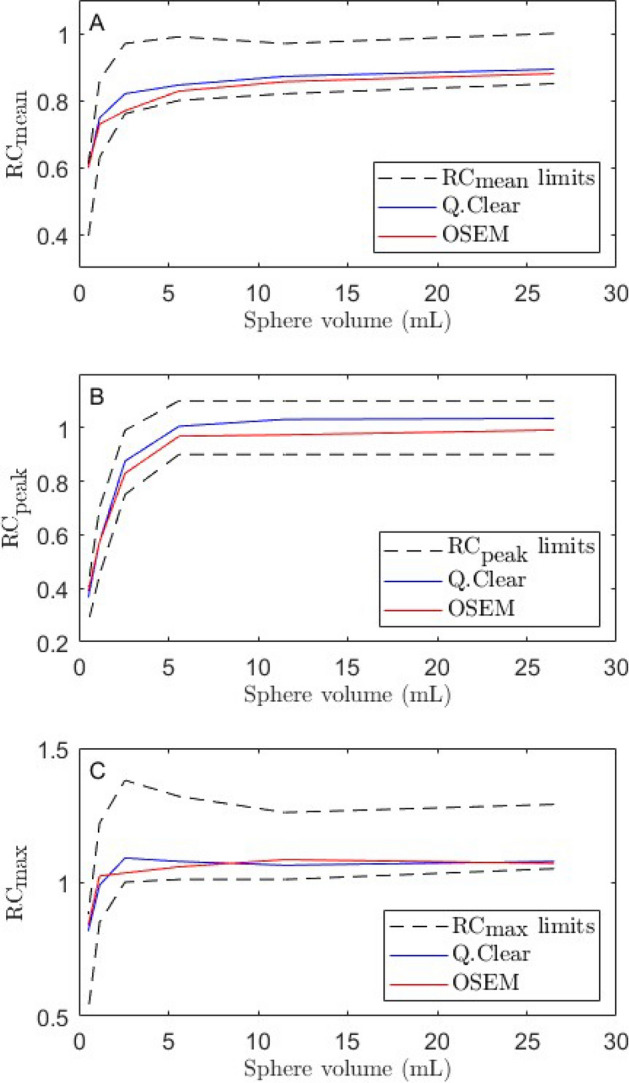
Recovery coefficients as a function of sphere size from the NEMA IEC phantom scans for the Q.Clear and OSEM reconstructed images complying with the EARL 2 standards for the (A) SUV_mean_, (B) SUV_peak_ and (C) SUV_max_

## Data Availability

The datasets used and/or analyzed during the current study are available from the corresponding author on reasonable request.

## A Reconstruction parameters

To ensure compliance with the EARL 2 standards, the $\beta$-factor for the Q.Clear reconstruction was set to 1200. For the standard OSEM reconstruction, the number of subsets equalled 17, the number of iterations equalled 3, the filter cutoff was set to 5 mm with the Z-axis filter set to ‘heavy’, The mean recovery coefficient (RC_mean_), peak recovery coefficient (RC_peak_) and maximum recovery coefficient (RC_max_) for both reconstruction methods are depicted in Fig. 6.

## B AIF parameters

For both reconstruction methods, the mean free parameters for the fit of the AIFs of the five study participants, as described in Eq. 1, are shown in Table 6.

**Table 6:** The mean value of the AIF fit parameters across the 5 study participants from the Q.Clear and OSEM reconstructed images

	Free parameter								
Reconstruction method	$a$ (min^−1^)	$b$	${A}_{1}$	${A}_{2}$	${A}_{3}$	${\lambda }_{1}$ (min^−1^)	${\lambda }_{2}$ (min^−1^)	${\lambda }_{3}$ (min^−1^)	$\tau$ (min)
Q.Clear	58.1 $\pm$ 16.4	− 30.7 $\pm$ 14.6	8.69 $\pm$ 2.29	1.43 $\pm$ 0.32	0.509 $\pm$ 0.075	5.24 $\pm$ 1.50	0.217 $\pm$ 0.058	0.0249 $\pm$ 0.0067	0.675 $\pm$ 0.139
OSEM	41.7 $\pm$ 16.4	− 20.8 $\pm$ 11.6	7.76 $\pm$ 1.56	1.33 $\pm$ 0.22	0.433 $\pm$ 0.088	4.36 $\pm$ 0.87	0.181 $\pm$ 0.023	0.0235 $\pm$ 0.0080	0.658 $\pm$ 0.126

## C Pharmacokinetic modelling

To determine the impact of the two reconstruction methods on the bias of the ^18^F-FDG influx rate compared to a gold standard, artificial TACs of regions with a set ^18^F-FDG influx rate were generated. Under the assumption of irreversible ^18^F-FDG uptake in tissue, the concentration of ^18^F-FDG in the tissue over time is given by [19]:

5 $$C_{t} \left( t \right) = \left( {\frac{{K_{1} k_{2} }}{{k_{2} + k_{3} }}e^{{ - \left( {k_{2} + k_{3} } \right)t}} + \frac{{K_{1} k_{3} }}{{k_{2} + k_{3} }}} \right) \otimes AIF\left( t \right)$$

Where ${K}_{i}$ [ml·min^−1^·g^−1^], ${k}_{2}$ [min^−1^] and ${k}_{3}$ [min^−1^] are the rate constants of the tissue and ${C}_{t}\left(t\right)$ [kBq/ml] is the ^18^F-FDG concentration in the tissue as a function of time.

The corresponding ^18^F-FDG influx rate ${K}_{i}$ of the tissue with rate constants ${K}_{1}$, ${k}_{2}$ and ${k}_{3}$ is given by:

6 $$K_{i} = \frac{{K_{1} k_{3} }}{{k_{2} + k_{3} }}$$

## Abbreviations

AIF: Arterial input function
BSREM: Blok sequential regularized expectation maximization
MC: Monte Carlo
METC: Medical ethics review committee
OSEM: Ordered subset expectation maximization
SAFOV: Short-axis field-of-view
SAT: Subcutaneous adipose tissue
TAC: Time activity curve
VOI: Volume of interest
